# Analysis of cognitive and attentional profiles in children with and without ADHD using an innovative virtual reality tool

**DOI:** 10.1371/journal.pone.0201039

**Published:** 2018-08-15

**Authors:** Débora Areces, Julie Dockrell, Trinidad García, Paloma González-Castro, Celestino Rodríguez

**Affiliations:** 1 Department of Psychology, University of Oviedo, Oviedo, Spain; 2 Department of Psychology, University College London, London, United Kingdom; Georgetown University, UNITED STATES

## Abstract

In previous studies, children with Attention-Deficit Hyperactivity Disorder (ADHD) have been found to have more difficulties with processing speed, working memory, and attentional tasks. The present study aimed to compare the cognitive variables (working memory and processing speed) and the attentional profiles of a sample of students with and without ADHD, using scales from the WISC-IV, and the virtual reality-based attentional test known as *‘Aula Nesplora’*; and determine the extent to which the aforementioned variables may predict student group membership. A total of 88 students took part in this study (66 males and 22 females), aged from 6 to 16 years (*M* = 10.20; *SD* = 2.79). The sample was divided into two groups: an ADHD group (*n* = 50) and a Control group (*n* = 38). Students in the ADHD group obtained lower scores in working memory and in processing speed, as well as demonstrating poorer performance in Aula Nesplora than did their peers. Working memory, and the number of omissions, were both shown to be reliable predictors of group membership. This study revealed the importance of obtaining data from attentional variables differentiated by modality when considering cognitive variables, in order to better characterize the difficulties experienced by individuals diagnosed with ADHD.

## Introduction

ADHD (Attentional Deficit and Hyperactivity Disorder) is a common neuropsychiatric disorder in childhood with prevalence rates ranging from 5 to 7% in the school-age population [1]. According to the American Psychiatric Association [2] children with ADHD experience high levels of overactivity, impulsivity, and inattention. Three subcategories or presentations of the disorder can be distinguished according to these symptoms: the combined presentation, the predominantly inattentive presentation, and the predominantly impulsive/hyperactive presentation.

Generally, the characteristic symptoms of ADHD have serious consequences which result in difficulties in scholastic, social and familial contexts [3, 4]. Specifically, children with ADHD have a higher probability of repeating a grade and completing fewer grades at school than children without ADHD. Moreover, dropping out of high school is three times more likely among youth with ADHD [5].

Nowadays, some children may be evaluated by clinicians using only a single information source, such as a semi-structured interview with the patient and/or parents. In this sense, comprehensive assessments are generally preferred over single-reports because they allow us to understand the children´s difficulties and offer the opportunity to rule out alternative explanations for the pattern of symptoms. Within the context of these assessments, cognitive and neuropsychological measures facilitate an unbiased evaluation of the core problems [6].

Considering the importance of carrying out an objective assessment, the attentional profile of children with ADHD has been widely studied by means of Continuous Performance Tests (CPTs), which provide quantitative data on different attentional variables of interest and have been shown to be useful in the diagnosis of ADHD [7]. A number of CPT studies, as well as a meta-analytic review of CPT research, have indicated that ADHD-related poor performance can be identified by measuring omission and commission error rates in ADHD cohorts and comparing these to matched control groups [8]. In this sense, errors of omission (such as the absence of a response to a target stimulus) are assumed to reflect symptoms of inattention, while errors of commission (mistaken responses) are assumed to reflect symptoms of impulsivity [8, 9]. Moreover, these types of tests are frequently used to reveal the treatment-efficacy of methylphenidate, thereby proving useful in identifying statistically significant reductions in the rates of both error types (omissions and commissions) [10].

Nonetheless, these types of tests are commonly criticized for their low ecological validity [8, 11]. The symptoms of ADHD not always are revealed when a child is performing a neutral task in a small room, with a single adult, and under controlled contextual conditions, as often happens in testing situations. Moreover, many authors [11, 12] sustained that these assessment tools presented sufficient sensitivity to ADHD, but specificity was not adequate. Sensitivity consists on the ability of a test to identify the presence of a disorder, while specificity refers to the ability of a test to detect the absence of a disorder, in this case the absence of ADHD. In this sense, although ideally the CPTs should have high in both attributes, frequently the clinician must sacrifice the degree of one or the other such attribute, thus adding emphasis to false positive diagnostic errors (higher sensitivity) or to false negative errors (higher specificity) [12].

Efforts to find improved assessment methods that offer higher ecological validity, as well as better sensitivity and specificity levels, have led to new techniques for evaluating ADHD that are based on the use of Virtual Reality (VR). Nowadays, new technological developments in the field of VR have generated an innovative and interesting option for carrying out neuropsychological evaluations of many cognitive processes [13, 14]. By creating dynamic and immersive virtual-environments, VR has become a useful device for neuropsychological assessments of behavior and cognitions [15]. The main advantages of VR are that the experimental setting is much more controllable than in real life and various measures can be recorded simultaneously. Furthermore, it allows an objective laboratory-based assessment as demanded by researchers [16]. In this way, it is possible that VR-based neuropsychological assessments can provide comprehensive information for predicting the patient’s real-world functioning [17]. Moreover, several authors have examined the concurrent and construct validities and temporal stability of VR CPTs [18]. Similarly, VR CPTs have also been shown to be effective for revealing the effect of a pharmacological treatment in more realistic conditions than can be offered by traditional CPTs [19]. Other studies comparing traditional CPTs and VR CPTs have revealed evidence in favor of the diagnostic validity of a ‘Virtual Classroom’, because the measure has better discriminant validity with respect to distinguishing between ADHD children and healthy controls on all CPT parameters: total correct responses, the number of commission and omission errors and on reaction time to targets [20]. In particular, a recent study identified significant differences between performance in a virtual environment and a traditional one, with longer reaction times evident in virtual reality. This highlights the negative influence of auditory distractors on attention performance in cases of children with ADHD [18]. Similarly, other studies using VR-CPT have shown that children with ADHD differ from control subjects in terms of time-related effects on performances. While controls sustained their performance for longer time-periods during virtual reality tasks, children with ADHD showed significantly decreased performance over time [21].

These advances present a huge number of advantages over more traditional techniques [22]. For example, the patient is assessed in a more realistic environment [23]. Accordingly, Aula Nesplora represents an important innovation in the diagnosis of ADHD [24]. This novel test involves tasks of sustained attention and response inhibition, which take place in the context of a virtual classroom. Moreover, Aula Nesplora offers additional information which is very useful for intervention guidelines, as it provides attentional data which is differentiated by the sensory channels (visual and auditory), the type of task (x-go and x-no go tasks), and the presence or absence of distracters. In addition, the test provides a reliable indicator of motor activity during performance [25]. This information is important in determining the severity of ADHD, a key aspect of an ADHD diagnosis referred to in the DSM-5 [2], and also in providing insights into the modalities of presentation or type of tasks that the child can benefit from in further interventions. For example, by use of an Aula Nesplora report, clinicians are now able to identify whether a participant can concentrate better when the information is presented by the visual or the auditory channel. With regard to the effectiveness of Aula Nesplora, different studies have demonstrated that the Aula Nesplora test is not only useful for differentiating between ADHD and non-ADHD symptomatology [23], but it also useful for discriminating across the different types of ADHD presentations [26]. A recent study [26] found that the many additional variables provided by Aula Nesplora made it possible to distinguish predominantly Impulsive/Hyperactivity (I/H) and Combined presentations of ADHD from control group data, while also detecting the differences between the I/H and Inattentive presentations. However, differences between the Inattentive and Combined presentations were only identified when the Aula Nesplora test results relating to the auditory channel were considered.

Conversely, other neuropsychological literature considers that although CPTs (both traditional, and virtual) identify impairment in ADHD patients, no one test is sufficient (on its own) in making a diagnosis, considering that other types of variables have also been shown to be affected by ADHD morbidity [27, 28]. Several studies have also identified cognitive variables which are impaired in ADHD [29, 30]. In particular, comparisons of the cognitive profiles of children with ADHD and average intelligence, using the WISC-IV [31], have shown that children with ADHD perform more poorly on working memory and processing speed than on perceptual organization and verbal comprehension [31–34]. Similarly, further studies have indicated that the disorder is characterized by cognitive inefficiencies and/or by multiple specific deficits affecting several cognitive abilities [27]. In addition, other researchers have found that children with ADHD, Learning Disabilities and Autism typically present with impairments in working memory, processing speed and graphomotor skills [35]. Moreover, the American Academy of Child and Adolescent Psychiatry-AACAP-guidelines [36, 37] also state that psychological evaluation is not mandatory for ADHD diagnosis, but is however, recommended in patients with poor academic achievement or suspected low IQ levels.

Regarding processing speed, a large number of researchers support the view that a low score in processing speed is one of the best predictors of ADHD, especially for those with inattentive symptoms [38, 39]. Moreover, there is a great deal of evidence suggesting that deficits in processing speed affect more complex reading skills (e.g. reading fluency), which might explain the high co-occurrence of ADHD and Reading Disabilities [39, 40].

On the other hand, with respect to the Executive Functions, many authors have observed an impairment in working memory in children with ADHD [41–44]. Working memory is a limited-capacity system for temporarily storing and processing internally held information for use in guiding behavior [44]. Gallagher and Blader [45] highlighted that while children with ADHD share some neuropsychological features with children suffering from other mental disorders (e.g. schizophrenia, anxiety, depression), ADHD is associated with a particular neuropsychological profile, characterized (along with other problems) by specific impairments in working memory processes. Similarly, other authors [45,46] confirmed that impairments of various working memory components are present in children with ADHD.

Other researchers have focused on working memory as a key component, analyzing whether academic problems in ADHD are due to ADHD symptoms, working memory deficits, or both [47,48]. The results are mixed, with some authors affirming that working memory has a direct effect on academic performance [49,50], while others suggest that an impairment in working memory increases rates of internalizing/externalizing problems, which in turn, affect academic performance [50]. Internalizing problems are behaviors that usually cause internal distress such us anxiety and depression. In contrast, externalizing problems are behaviors that generate conflict with others, such us aggressive, rule-breaking and impulsive behavior [51].

These findings suggest that both processing speed and working memory are relevant neurocognitive markers of ADHD and other disorders which share common symptoms [33].

Given the high prevalence rates of ADHD and the low ecological validity of the majority of CPTs, virtual reality tasks provide a means of examining the relationship between performance and cognitive profiles in a more realistic environment. For this reason, considering that the field of assessment tools for detecting the ADHD is still in development, there is a need to continue research aimed at better understanding how the combination of cognitive and neuropsychological measures may facilitate an accurate diagnosis of this disorder. While previous studies have focused on processing speed, working memory and executive-function variables, the present study aimed to detect the effectiveness of all three variables in order to predict ‘real-world’ ADHD symptoms. For this reason, the study had two main objectives: 1) To describe and compare cognitive profiles provided by a cognitive scale (using working memory and processing speed scales from the WISC-IV) to behavioral profiles provided by a VR-CPT (using variables from the Aula Nesplora test) in a sample of students with and without ADHD; and 2) To determine the extent to which the aforementioned variables can predict student group membership, while also controlling for the potential explanatory effects of gender and age.

## Method

### Participants

A non-probabilistic sample of 88 students took part in this study, 66 males (75%) and 22 females (25%) between 6 and 16 years of age (*M* = 10.20; *SD* = 2.79). Of the total sample, 50 (56.8%) had a diagnosis of ADHD and 38 (43.2%) were controls. The average IQ (using standard scores) of the total sample was 110.05 (*SD* = 13.93). Only the following two scales from the WISC-IV were considered for inclusion criteria: Verbal Comprehension (*M* = 114.17; *SD* = 13.77), and Perceptual Reasoning (*M* = 108.37; *SD* = 15.48); as the remaining scales (Processing Speed and Working Memory) were the dependent variables in the present study. There were significant differences between the groups [processing Speed: *F*(1,87) = 4.607, *p* = .033, *ηp2 =* .102 ; working memory: *F*(1,87) = 9.633, *p* = .003, *ηp2 =* .051]. No statistically significant differences were found between the ADHD and Control groups regarding perceptual reasoning (*F*(1,87) = 2.868, *p* = .081, *ηp2 =* .032 ), verbal comprehension (*F*(1,87) = 3.799, *p* = .159, *ηp2 =* .042), and age (*F*(1,87) = 3.061, *p* = .806, *ηp2 =* .001). However, although there were no differences in the age ranges of the samples, age was included as a covariate in subsequent statistical analyses, in order to avoid omitting the explanatory effect of this variable within each diagnostic group.

Taking into account that children with ADHD usually show lower scores in working memory and processing speed than their peers [34, 38, 41, 42], and given that analyzing these differences in one of the goals of the present study, only the scales of perceptual reasoning and verbal comprehension from the WISC-IV were used as inclusion criteria. Thus, only students with an IQ between 80 and 130 (in perceptual reasoning and verbal comprehension) were included in the present sample.

### Inclusion criteria

The sample was recruited from children presenting at a hospital-based ADHD-diagnosis clinic due to their parents or teachers suspecting attentional and/or inhibitory control problems. They had been identified as fulfilling the DSM-5 criteria for ADHD [2] by mental-health professionals (typically one or more neuro-psychiatrists). I Subjects with a cognitive deficit, such as Asperger’s syndrome, Guilles de la Tourette syndrome, anxious depressive disorders were excluded from the study, as were those with comorbid behavioral/learning disorders. The schools attended by the participants were in urban and semi-urban zones from a region in the north-west of Spain.

A further control sample was recruited from the same schools to serve as a non-ADHD control group. Students were included in the control group if they had no reported history of behavioral or emotional problems in school or at home. Participants with a IQ below 80 and over 130 in these scales were excluded from the study. The participants came from families of medium socio-economic status.

Although students´ assignation to groups was made based on DSM-5 Criteria (APA, 2013), the EDAH scale [52] was completed by families (parents) in order to verify that students in the different groups differed on the scales of attention deficit and impulsivity/hyperactivity symptoms. Table 1 shows results from this analysis.

**Table 1:** ADHD symptoms in the diagnostic groups and differences (as per EDAH scale).

	Diagnostic groups		Total sample	Differences		
	ADHD	Control				
	*M(SD)*	*M(SD)*	*M(SD)*	*F*	*p*	*ηp*^*2*^
EDAH-AD Raw score	9.96 (2.473)	6.88 (3.193)	8.78 (3.214)	7.379	.009	.129
EDAH-I/H Raw score	8.78 (3.994)	6.00 (3.317)	7.44 (3.908)	15.281	< .001	.234
EDAH-ADHD Raw score	18.63 (5.108)	12.50 (5.605)	15.83 (6.061)	15.400	< .001	.235

Note. EDAH-AD = attention deficit symptoms; EDAH-I/H = impulsivity/ hyperactivity symptoms; EDAH-ADHD = attention deficit + impulsivity/hyperactivity symptoms.

ADHD (n = 50), Control (n = 38).

As Table 1 shows, there were statistically significant differences between the diagnostic groups in both attention deficit and impulsivity/hyperactivity symptoms. Students in the EDAH-ADHD group showed more symptoms in both scales, as was to be expected. When both EDAH-presentations were taken together, group differences remained constant. Moreover, Multivariate Analysis of Variance (of the ADHD group) revealed that there were no statistically significant differences (*p* = .065) between presentation-types measured by EDAH scale (inattention, impulsivity and hyperactivity)

None of the participants with ADHD were receiving pharmacological treatment at the time of evaluation, or during the assessment of their individual symptoms, as it was important to gauge the true (unmasked) severities of ADHD symptoms in order to ascertain their relationship to the resultant effects of ADHD, such as lower performance in processing speed, working memory and executive variables in comparison to children without this disorder.

### Instruments and measures variables

The following instruments and measures were used in the present study:

*The Wechsler Intelligence Scale for Children-IV (WISC-IV)* by Wechsler [31] was used as a measure of intelligence, expressed in terms of Intelligence Quotient (IQ). This scale can be administered to children and adolescents between the ages of 6 years and 16 years 11 months and provides a general estimation of subject IQ as well as four different measures related to different abilities. In the present study, the four components of working memory, processing speed, verbal comprehension and perceptual reasoning were used, whereas a measure of total IQ was not included. The focus of the study was on specific abilities rather than general intellectual capacity. The standard scores of working memory and processing speed were included as dependent variables in the present study, while IQ in verbal comprehension and perceptual reasoning were used for sample inclusion purposes. Cronbach´s Alpha of the WISC-IV was .677 in the current sample.

*Aula Nesplora* [24] was administered in order to determine participants’ attentional profile. This is a Continuous Performance Test (CPT) based on a virtual reality environment that reproduces the conditions of a regular classroom. Aula Nesplora evaluates attention, impulsivity, processing speed, and motor activity in children and adolescents between 6 and 16 years of age. The virtual reality environment is shown through 3D glasses (Head Mounted Display, HMD). Motion sensors and headphones are also included in order to make the task as realistic as possible. The participant takes the perspective of a student sitting in one of the desks looking at the blackboard. Head movements are registered by sensors located in the glasses; thus, the software updates the angle of vision, giving the subject the feeling of actually being in a virtual classroom (an image of the AULA Nesplora hardware is provided in S1 Fig).

The duration of Aula Nesplora test lasts for 20 minutes. The test consists of three phases that are gradually explained by a virtual teacher (See S2 Fig). The objective of the first phase is to immerse the participant in the context of virtual reality, and it consists of visually locating balloons and popping them. Below this scene is a task based on the “x-no” paradigm (traditionally known as “no-go”) in which the participant must press a button provided that he or she does not see or hear the stimulus “apple.” Finally, an “x” paradigm (or “go”) is also incorporated, with participants being asked to press a button whenever they see or hear the number “seven”. Thus, not only the delivery response but also its inhibition is assessed.

As described in the studies of Díaz-Orueta et al. [25], the features of Aula Nesplora parameters present some differences to other CPTs due to the more complex nature of stimuli present in this test. Therefore, while the time of exposure for visual stimuli is 250 ms, for auditory stimuli the mean time is 650 ms, as the exposure is a function of the length of each word being presented (e.g. ranging from 470 ms for the shortest word, to a maximum of 891 ms). In addition, once the auditory or visual item is presented, the child has a maximum of 2500 ms to press the button and thus register his or her answer into the frame of the presented stimulus. The total number of the Aula Nesplora items presented is 360 (of which 180 are ‘targeted stimuli’, and 180 are ‘non-targeted’ stimuli).

Regarding the distribution of the distractors, it is different depending on the type of task (“x-no”, or “x”). During the first task (“x-no” items) there are 9 distractors (two visual, three auditory and four combined). However, in the second task (“x” items) there are 7 distractors (two visual, three auditory and one combined).

The variables provided by the instrument do not differ from those of other CPTs regarding attention deficit and hyperactivity/impulsivity measures (omissions, commissions, response time, and motor activity); however, they complement this information, by differentiating the measures of sensory modality (visual vs. auditory), presence/ absence of distractors, and task type (go vs. no-go), thereby leading to different execution profiles. High scores in these measures are related to attentional deficits. Both the general and specific measures (variables analyzed by different conditions) have been shown to provide different contributions to the explanation of inattention and hyperactivity/impulsivity symptoms in ADHD [26], thus analyzing each of the conditions separately is pertinent. Raw scores in omissions, commissions, response times and motor activity for the entire task (general measures), as well as per each of the six conditions individually, were included as dependent measures in the present study. The only exception to this related to visual and auditory stimulation conditions, as a measure of motor activity is not be provided. Cronbach´s Alpha in this sample was .621.

*The Scale for the assessment of Attention Deficit Hyperactivity Disorder (EDAH)* [52] was completed by families (children´s parents). It consists of 20 items that provide information on the presence of symptoms related to attention deficit and hyperactivity/impulsivity. It differentiates between ADHD and control groups, as well as between ADHD presentations. The following variables were included in the present study: EDAH-AD (score in the items that measure Attention Deficit), EDAH-I/H (score in Impulsivity/Hyperactivity items), and EDAH-ADHD (the sum of attention deficit plus Impulsivity/Hyperactivity symptoms). The reliability of the instrument, using Cronbach´s Alpha, is high for the whole scale (.929) and its components: DA (.898), H (.849), and CD (.899). Cronbach´s Alpha was .855 in the current sample.

### Procedure

**A** This study was specifically approved by the CEIC Ethics Committee of the Principality of Asturias (Approval No. CPMP/ICH/135/95. CODE: TDAH-Oviedo). The clinical database used for this study is available (see S1 Database) in accordance with The Code of Ethics of the World Medical Association (Declaration of Helsinki) [53]. All subjects and their parents gave written informed consent after receiving a comprehensive description of the study protocol. Participants had volunteered to be involved in this study and they were not given any incentive to take part in it. To that end, once parental consent to evaluate the children was provided, the corresponding tests were conducted to verify the diagnosis and to participate in this research.

Following the above recruitment procedure, participants underwent two cognitive tests (administered by the present study’s psychologists): 1) The Wechsler Intelligence Scale for Children-IV [31]; and 2) The Aula Nesplora Test [24]. The two tests were individually administered to each child during two separate assessment sessions.

### Data analysis

This study analyzed the differences in cognitive and attentional variables between two groups of students with and without ADHD and examined the discriminant value of these variables in predicting group membership. In order to accomplish that, data analyses were conducted in three steps:

First, the descriptive statistics for the variables under study were analyzed, paying special attention to skewness and kurtosis. Kline's criterion [54], according to which the maximum scores accepted for skewness and kurtosis range between 3 and 10, was used. As the results showed that cognitive variables from the Wisc-IV had met this criterion (Table 2), parametric analyses were then carried out. With respect to the attentional variables, some of the results did not meet this criterion, particularly commissions in the ADHD group and omissions in the control group, therefore non-parametric analyses were conducted (Table 3).

**Table 2:** Differences between groups and descriptive statistics for the dependent variables (Wisc-IV).

	Diagnostic groups						Differences	
	ADHD			Control				
	*M(SD)* *IQ*	*K*	*S*	*M(SD)* *IQ*	*K*	*S*	*F*	*ηp*^*2*^
Cognitive variables								
WM	103.16 (12.321)	.845	.164	111.447 (12.564)	.640	.272	9.603**	.100
PS	98.10 (13.158)	-.173	.276	103.89 (13.210)	-.398	.088	4.092*	.045

*Note*. M = mean; SD = standard deviation; IQ = intellectual quotient for the variables from Wisc-IV; K = Kurtosis; S = Skewness; WM = Working memory; PS = Processing Speed.

ADHD (n = 50), Control (n = 38).

*p< .05.

**p< .005.

***p< .001.

**Table 3:** Differences between groups and descriptive statistics for the dependent variables (Aula Nesplora).

	Diagnostic groups						Differences		
	ADHD			Control					
	*M(SD)* *Raw score*	*K*	*S*	*M(SD)* *Raw score*	*K*	*S*	*U*	*Z*	*r*
Aula Nesplora General									
O	41.06 (29.391)	-1.121	.514	14.68 (1.960)	12.472	2.937	392.000	-4.703 ***	.501
C	19.20 (18.325)	14.496	3.363	12.63 (7.521)	.266	.865	692.000	-2.176 *	.232
RT	954.197 (145.446)	.375	.002	854.333 (111.187)	1.012	.785	524.000	-3.572 ***	.380
MA	.799 (.617)	2.665	1.337	.441 (.287)	.740	1.084	620.500	-2.776 **	.296
Aula Nesplora Visual Channel									
O	27.42 (21.017)	-.900	.625	10.71 (10.076)	12.466	3.045	455.500	-4.169 ***	.444
C	10.20 (9.491)	11.309	2.873	7.26 (4.209)	.854	1.009	770.500	-1.517	.161
RT	808.342 (146.187)	.298	.297	711.969 (129.560)	.810	1.049	539.000	-3.462 **	.370
Aula Nesplora Auditory Channel									
O	13.64 (16.278)	4.820	2.190	3.97 (3.605)	1.039	1.195	457.500	-4.164 ***	.444
C	9.00 (9.647)	13.243	3.298	5.37 (4.253)	-.364	.815	681.000	-2.273 *	.242
RT	1091.088 (152.135)	.643	-.176	1000.307 (046)	.288	.323	585.000	-3.075 **	.328
Aula Nesplora X-Tasks									
O	70.58 (23.896)	.326	1.020	52.774 (19.925)	1.303	1.256	500.500	-3.809 ***	.406
C	8.24 (5.384)	15.617	4.168	2.29 (3.179)	3.483	2.002	557.000	-3.345 **	.356
RT	1040.879 (186.796)	-.479	.250	943.652 (138.989)	-.253	.331	675.000	-2.317 *	.247
MA	.957 (.771)	4.514	1.600	.506 (.335)	.302	.928	602.000	-2.932 **	.312
Aula nesplora X-no Tasks									
O	34.02 (25.989)	-.898	.632	12.00 (12.460)	14.171	3.133	416.000	-4.502 ***	.480
C	10.96 (5.897)	.270	.453	10.34 (5.181)	-.101	.200	916.000	-.287	.003
RT	932.789 (149.410)	.710	.033	831.425 (111.296)	1.073	.779	818.000	-3.639 ***	.388
MA	.606 (.509)	2.842	1.553	.348 (.268)	2.261	1.642	645.000	-2.566 **	.273
Aula Nesplora Distractors									
O	15.38 (11.597)	-.204	.836	5.89 (5.321)	6.987	2.159	434.500	-4.352 ***	.464
C	6.50 (5.358)	5.238	1.915	5.50 (3.311)	.141	.642	894.000	-.474	.005
RT	967.650 (154.926)	.960	-.142	852.341 (117.145)	1.108	.818	473.000	-4.018 ***	.428
MA	.767 (.600)	2.100	1.254	.455 (.294)	.071	.989	680.000	-2.275 *	.242
Aula Nesplora No distractors									
O	25.68 (18.870)	-1.092	.527	8.79 (9.257)	12.306	2.972	388.000	-4.739 ***	.505
C	12.70 (13.606)	16.646	3.623	7.13 (4.839)	.206	1.015	616.000	-2.820 **	.300
RT	945.213 (148.947)	-.187	.238	856.168 (114.666)	1.014	.738	594.000	-2.999 **	.320
MA	.885 (.682)	2.955	1.401	.478 (.311)	1.360	1.187	596.000	-2.978 **	.317

*Note*. M = mean; SD = standard deviation; K = Kurtosis; S = Skewness; O = omissions; C = commissions; RT = response time associated with a correct answer; MA = motor activity during the task (this measure is not provided for visual and auditory channels).

ADHD (n = 50), Control (n = 38).

*p< .05.

**p< .005.

***p< .001.

Second, in the case of the cognitive variables (working memory and processing speed), separate univariate analyses of covariance (ANCOVAs) were performed to analyze the differences between the groups, using age as a covariate. Regarding attentional variables, the Man-Withney U test was carried out for each dependent variable. The first analysis was performed using the total scores in the test (omissions, commissions, response time and motor activity). Subsequently, six separate analyses for the different conditions offered by the test (visual vs. auditory channel; and X vs. no-X task; presence vs. absence of distractors) were performed, in order to determine which of these conditions generated the greatest differences between the groups. For these statistical analyses, the diagnostic group was the independent variable.

Rosenthal`s r statistic [55] was used as a measure of effect size in the case of non-parametric analyses, while for the cognitive variables, an η^2^ of parametric analysis was performed [56]. Effect size was interpreted following Cohen’s [56] criteria, which define a small effect size as η2 = .010 (Rosenthal`s r = .10), a medium effect size as η2 = .059 (Rosenthal`s r = .30), and a large effect size as η2 = .138 (Rosenthal`s r = .50).

Finally, once the existence of statistically significant differences between the groups was verified (Table 2), different discriminant analyses were conducted to determine the specificity and sensitivity levels of each dependent variable (cognitive and attentional variables) in identifying subjects in each group. Four discriminant analyses were performed: the first analysis with the general scores of Aula Nesplora, and other three for each pair of test conditions. Thus, visual vs. auditory channel, X vs. X-no task, and presence vs. absence of distractors were grouped in pairs, as MANCOVAs revealed a quite similar pattern of differences regarding each component of the dyad (Table 3). Age and gender were also included in the analyses to determine their potential discriminant value. Age was recoded as a dummy variable for these analyses. Since significant but not too high correlations between variables is an important criterion to conduct discriminant analysis, Pearson´s correlations between attentional and cognitive measures were calculated, using Aula Nesplora general measures. SPSS 19 [57] was used in the analysis of data, establishing p < .05 as the criterion for statistical significance.

## Results

### Differences in cognitive and attention variables between students with and without ADHD

#### Cognitive measures

Table 2 shows descriptive statistics of the cognitive variables from the Wisc-IV, results using ANCOVAs, taking into account age as covariate. The ANCOVAs indicated that both working memory and processing speed standard scores differed statistically significantly between the diagnostic groups. The control group showed higher scores (better performance) than the ADHD group in the cognitive variables, and effect size was larger for working memory than for processing speed.

As shown in Table 2, students without ADHD systematically showed higher IQ scores in working memory and processing speed, although effect size was considerably lower in the case of the second variable.

These scales correlated significantly with each other, *R*^*2*^ = .384, *p* < .001 and with the scales of verbal comprehension and perceptual reasoning. Specifically, working memory correlated positively and significantly with both the additional scales [*R*^*2*^ = .223, *p* < .05, and *R*^*2*^ = .271, *p* < .05 for verbal comprehension and perceptual reasoning, respectively], while processing speed only correlated significantly (also positively) to perceptual reasoning, *R*^2^ = .333, *p* < .01. The correlation between verbal comprehension and perceptual reasoning was .609 (*p* < .01).

#### Attentional measures

Results from Aula Nesplora general measures (raw scores), as well as for each of the six conditions of the test are presented in Table 3. As can be observed, there was significant variability among students in the studied variables. It is worth noting in this sense that both ADHD and control groups show a considerably higher number of omissions than commissions in general and across the different conditions. Means indicate that the group with ADHD shows a greater amount of omissions and commissions, as well as longer response times and higher motor activity, than the group without ADHD. These differences are statistically significant in most of the cases.

#### Aula Nesplora general

In terms of the raw scores of general measures, the Mann-Withney U test indicated the existence of statistically significant differences between groups in all the dependent variables (see Table 3 for significance-values). The variables omissions and response time showed the greatest effect sizes (Table 3).

#### Aula Nesplora by condition

Concerning stimulation channels, the Mann-Withney U test indicated the existence of significant differences between groups in both channels (see Table 3). Effect size was similar in both channels, with the highest effects being in relation to omissions and response time. Thus, taking into consideration each variable separately, omissions and response time generated the greatest differences for both conditions.

With regard to the X vs. X-No condition, statistically significant between the groups were obtained in all the dependent variables, with the exception of commissions in the X-NO condition (Table 3). The variables showed similar effect sizes in both conditions. Again, the variables omissions, followed by response time, were the components which systematically generated the biggest difference between groups. It is also worth noting the effect of commissions, but only in the X condition, which reached an important effect size (> .35).

Finally, considering the presence or absence of distractors, the Mann-Withney U test showed the existence of statistically significant differences between students with and without ADHD in all the dependent variables, with the one exception again being commissions in the presence of distractors (Table 3). While commissions were less frequent in the group without ADHD in both conditions, significant differences between the groups were only found in absence of distractors. The distractors condition showed slightly higher effect sizes than the no-distractors condition, especially in relation to omissions, with which resulted in a r value exceeding .50. Consistent with the study’s findings concerning the distracters condition, omissions and response time were also the most significant variables in general terms. Although there were no statistically significant differences in age, the analysis revealed an age-related trend which affected AULA Nesplora performance. The figure (see S3 Fig) shows the age effect on significant AULA Nesplora variables, as confirmed by the Kruskal-Wallis test: Omissions, χ^2^(10) = 27.033, *p* = .003; and response time, χ^2^(10) = 27.159, *p* = .002.

#### Discriminatory value of cognitive and attentional variables identifying students with and without ADHD

First of all, correlation analyses revealed the existence of statistically significant associations between cognitive and attentional measures, but only in the case of working memory. This variable correlated negatively with all the Aula Nesplora general measures, being significant in the case of omissions, *R*^*2*^ = -.224, *p* < .05, and response time, *R*^*2*^ = -.217, *p* < .05.

Table 4 shows results from the different discriminant analyses, whose objective was to examine the explanatory power of the attentional and cognitive variables to predict student group membership (ADHD or Control group). To this end, attentional variables provided by Aula Nesplora were used (Aula Nesplora general measures, and separated analyses for pairs of conditions). Additionally, WISC-IV working memory and processing speed were also included as potential predictors of group membership, along with age and gender. Standardized coefficients represent the correlations between the discriminant function and the variables, revealing the most influential variable in each case. Function coefficients provide the resulting discriminant function.

**Table 4:** Results of discriminant analyses, using stepwise method. Analyses with Aula Nesplora general variables, and for pairs of conditions.

	Standardized Coefficients	Function Coefficients	*F*
Aula Nesplora General			
O	1.193	.050	26.077
Age	.610	.217	17.444
Constant		-3.690	
Aula Nesplora Visual vs. Auditory Channel			
O (Visual)	.798	.045	19.173
O (Auditory)	.525	.042	13.021
Age	.515	.183	10.807
WM	-.359	-.029	9.758
Constant		-.092	
Aula Nesplora X vs. X-no			
O (X-no)	.832	.039	23.181
WM	-.498	-.040	15.199
Constant		3.315	
Aula Nesplora Distractors vs. No distractors			
O (No distractors)	.838	.054	25.694
Age	.696	.248	16.882
RT (Distractors)	.462	.003	13.044
C (No distractors)	.429	.040	11.419
Constant		-6.966	

Note. WM = Working memory; O = omissions; C = commissions; RT = response time associated with a correct answer).

All models are significant at a p < .001 level. Only the variables that resulted statistically significant are shown.

Considering the first model (with Aula Nesplora general measures), only omissions and age were statistically significant predictors of group membership. Omissions showed the highest standardized coefficient, being the most relevant variable identifying subjects with and without ADHD. The statistics indicated that the older the student and the higher the score in omissions, the higher the probability to present ADHD. This model classified 76.1% of the sample correctly (66% from the control group, and 89.5% from the ADHD group).

Taking into consideration the different conditions of the task, omission was present in all the models tested, being the strongest predictor of student group membership. This variable was significant in both visual and auditory conditions, as well as in X-no task and under the no distractors paradigm, showing the same relationship to ADHD symptoms as in the previous model. Age was also a significant predictor in most of the models, showing in all of them a positive relationship with the presence of ADHD. Working memory turned was a significant predictor of group membership only in some test conditions (i.e., Visual vs. Auditory channel, and X vs. X-no task), but was not for the general measures, or when the condition Presence vs. Absence of distractors was taken into account. Finally, when the last pair of conditions were analyzed, two new variables were significant (i.e. response time under the distractors condition and commissions under the no distractors condition). These three models classified correctly a similar number of students as the model with general measures: the models with both Visual vs. Auditory channel and X vs. X-no task classified correctly 75% of the sample (66% and 64% of the controls, and 86.8% and 89.5% of the students with ADHD, respectively), while in the case of the Distractors vs. No distractors condition, 76.1% of the students were correctly classified (70% from the control group, and 84.2% from the ADHD group).

On the whole, the results indicated that the models that best classified students with ADHD were the models which consisted of the general measures of attention, and the one model that related to X vs. X-no conditions. In this way, the more parsimonious model was the first model which provided similar sensitivity and specificity values than the other one. On the other hand, the model that best identified students without ADHD was the last model, which included the Distractors vs. No Distractors condition. The current results demonstrated the important roles of omissions, age, and working memory deficit in predicting the probability of a child receiving a diagnosis of ADHD.

## Discussion and conclusions

The objectives of this study were: 1) To describe and compare cognitive profiles provided by a cognitive scale (using the working memory and processing speed scales from the WISC-IV) to behavioral profiles provided by a VR CPT (using the variables from the Aula Nesplora test) in a sample comprising students with and without ADHD; and 2) To determine the extent to which the aforementioned variables can accurately predict a student’s pre-determined diagnostic-group association.

In line with previous studies, the two groups of children differed significantly on the basis of their cognitive and behavioral profiles [7, 11, 17, 34]. At the cognitive level, children with ADHD showed lower scores in working memory and processing speed than the group without ADHD, although greater effect sizes between the groups were found in working memory, rather than in processing speed. These results are consistent with previous studies which have demonstrated how children with ADHD perform worse in processing speed and working memory tasks than their peers without ADHD [32, 33].

At the behavioral level, students with ADHD showed poorer performance in Aula Nesplora (more frequent omissions and commissions, higher response times and motor activity) than the control group. On the other hand, when the results were analyzed according to the type of task, both groups (ADHD and Control) produced more omissions as well as higher motor activity in Go-tasks. Given its design, Aula Nesplora provides the possibility of obtaining different executive profiles, which are also displayed under different contextual and stimulatory conditions. These differences may have important implications for the design and implementation of more adaptable interventions based upon broader-scoped profiles. More specifically, the results from such tests can show if a student concentrates better, and has more inhibitory control, is related to whether the stimuli are presented via auditory or visual channels. This type of information may be very relevant with respect to recommendations about how a study might obtain better results (e.g. whether to use more auditory channeling or to use more visual-based information through diagrams). Similarly, VR CPT also offers the possibility of observing the effect of ‘distractors’ upon student performance. This additional information is also useful for making other types of recommendations, such as the need of some students to be situated in a quiet place to perform tasks optimally.

This study’s findings are in agreement with the results obtained by Iriarte et al. [23], which also showed that omissions and response times were the variables which identified the largest differences between diagnostic-groups, and better predicted group-associations, by allowing for a greater degree of discriminant analysis. Moreover, this pattern remained when each specific condition was analyzed (e.g., presence and absence of distractors, visual and auditory channel, go/no go tasks). The relevance of these variables in relation to inattentive symptoms (as reported in previous studies [27]) may well be explained by the high prevalence of attention deficit symptoms reported in the current sample of students with ADHD. Nonetheless, separate analysis by modality showed that the statistical power of the test in discriminating students with and without ADHD was higher for the visual channel, for x-no tasks, and in presence of distractors. These results demonstrate the importance of collecting the same variables under different conditions in order to obtain an accurate diagnosis, while also increasing ecological validity by means of novel assessment tools [11]. The Aula Nesplora, unlike other traditional CPTs, provides the possibility of discriminating between tasks in both the presence and absence of distractors, an aspect which is relevant in the diagnosis of ADHD [23, 25] and important for the improvement of classroom learning environments. In particular, this study showed that response time variables collected in the presence of distractors are more effective in distinguishing between children with ADHD and children without ADHD, as the effect size demonstrated. These findings are in agreement with previous research [18] highlighting the negative influence of auditory distractors on attention performance in children with ADHD. The same study also showed significant differences between performance in the virtual environment and traditional CPTs. with longer reaction times in virtual reality. These results represent the advantages of assessing ADHD symptoms using VR instead of traditional CPTs, as VR allows the measurement of executive variables in the presence of far more realistic distracters.

Pertinently however, although both cognitive and attentional components were important in order to establish a profile in ADHD, the amount of variance explained by attentional variables was larger than that explained by the cognitive variables in the present study, as was indicated by the effect sizes. This means that although cognitive profiles in ADHD can be used to provide a diagnosis of ADHD, they may be insufficient in establishing a definitive diagnosis if utilized as an exclusive diagnostic tool. [33].

Concerning the study’s second objective, the purpose of which was to determine the extent to which attentional and cognitive variables can predict student diagnostic-group associations, the results showed that the specific attentional and cognitive variables analyzed allowed for the classification of a significant proportion of the students into diagnostic groups. In particular, omissions, working memory and age, were revealed as the most significant predictors within the different models analyzed. In this sense, working memory impairments, an increase in age, and the presence of a great number of omissions in Aula Nesplora were significantly related to a higher likelihood of having a diagnosis of ADHD. The models were more useful in discriminating subjects with ADHD than controls, as was expected, given that the nature of the task was aimed at identifying students with the disorder. In this sense, it was observed that, similar to other CPTs, this virtual reality tool has been demonstrated to have better levels of sensitivity than specificity [11, 12]. These results are consistent with previous studies that have identified the existence of a strong relationship between ADHD and working memory impairment. Highlighting how working memory affects students’ performance on attentional tasks. Some of them suggest that poor performance in attentional tasks and CPTs may not only be due to attentional problems, but also may be explained by working memory impairment [32, 33, 35]. Findings from the present study also demonstrate the importance of age in the diagnosis of ADHD. This relationship between age and performance on attentional tasks was predicted, as previous research suggests that the persistence of ADHD symptoms over time, might be an important indicator regarding the both severity of ADHD, and its presentation-subtype [43]. Similarly, other authors [22] have explained that while children without ADHD evidenced sustained performances over time in the virtual reality task, children with ADHD manifested a significant deterioration in performance during the test.

Finally, these findings suggest that, focusing only on one of the test conditions (e.g. Visual vs. Auditory channels) may have some advantages over the whole test, such as a reduction in data interpretation. However, this study also highlights the necessity of taking into account additional measures (working memory, for instance) in order to reach a similar discriminant value than that provided by the whole test itself and age, a model that, in turn, results more parsimonious. This would ultimately depend upon professionals´ choice and assessment purposes. Although this study suggests preliminary evidence supporting this hypothesis, additional studies are necessary.

### Limitations and future directions

Some limitations of the present study should be considered in future investigations. Firstly, additional studies with a wider sample size are needed in order to examine whether the statistical power of the variables analyzed is similar to that obtained in the present study. It is important to verify if these specific findings could be replicated in future researches. In addition, in order to identify the developmental processes of ADHD symptoms, it is important to analyze the statistical power of the variables in the different age groups. Finally, a direct comparison between the Aula Nesplora with other traditional CPTs would test the benefits of the use of ecologically valid tools in the ADHD diagnosis.

## Supporting information

S1 Fig 3D virtual glasses of AULA Nesplora test. The 3d virtual glasses allows to obtain a motor activity indicator to value the possible hyperactivity problems.
(JPG)

S2 Fig Virtual classroom provided by AULA Nesplora test. Virtual classroom environment where the patient does the tasks which are explained by a virtual teacher.
(TIF)

S3 Fig Age effect on AULA Nesplora variables. (TIF)

S1 Database Database used for the present study. (SAV)
