# Supplementary figures and images for: Analysis of cognitive and attentional profiles in children with and without ADHD using an innovative virtual reality tool
Source: PLoS One. 2018 Aug 15;13(8):e0201039. doi: 10.1371/journal.pone.0201039 (PMC6093610; doi:10.1371/journal.pone.0201039)

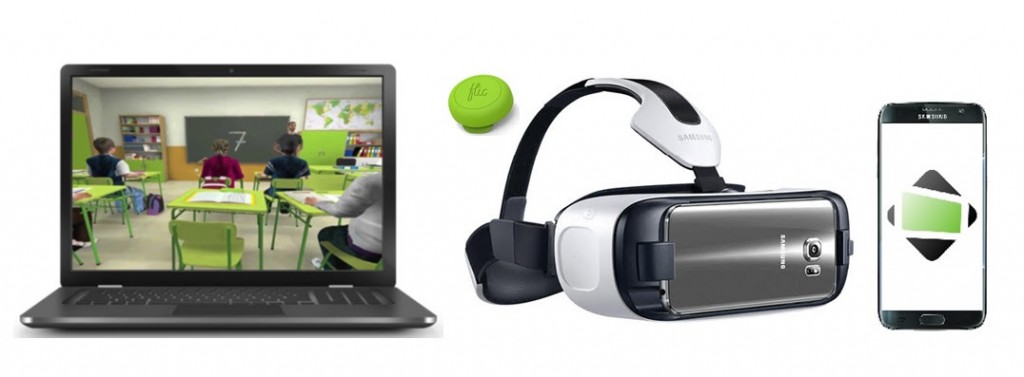

Supplement: S1 Fig — The 3d virtual glasses allows to obtain a motor activity indicator to value the possible hyperactivity problems. (JPG) [file pone.0201039.s001.jpg]

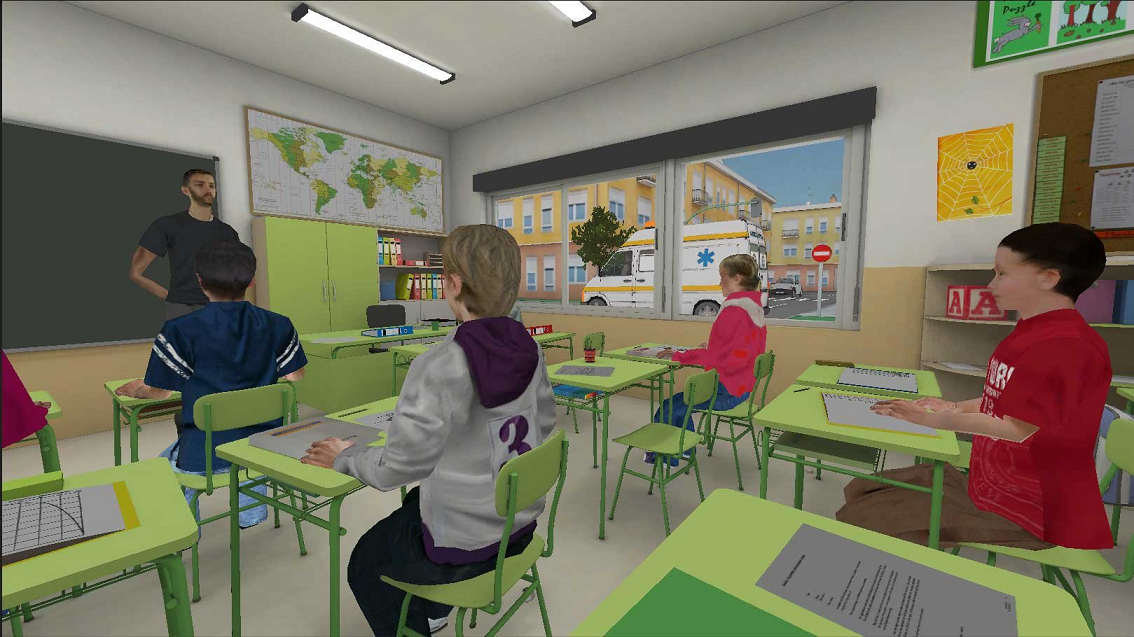

Supplement: S2 Fig — Virtual classroom environment where the patient does the tasks which are explained by a virtual teacher. (TIF) [file pone.0201039.s002.tif]

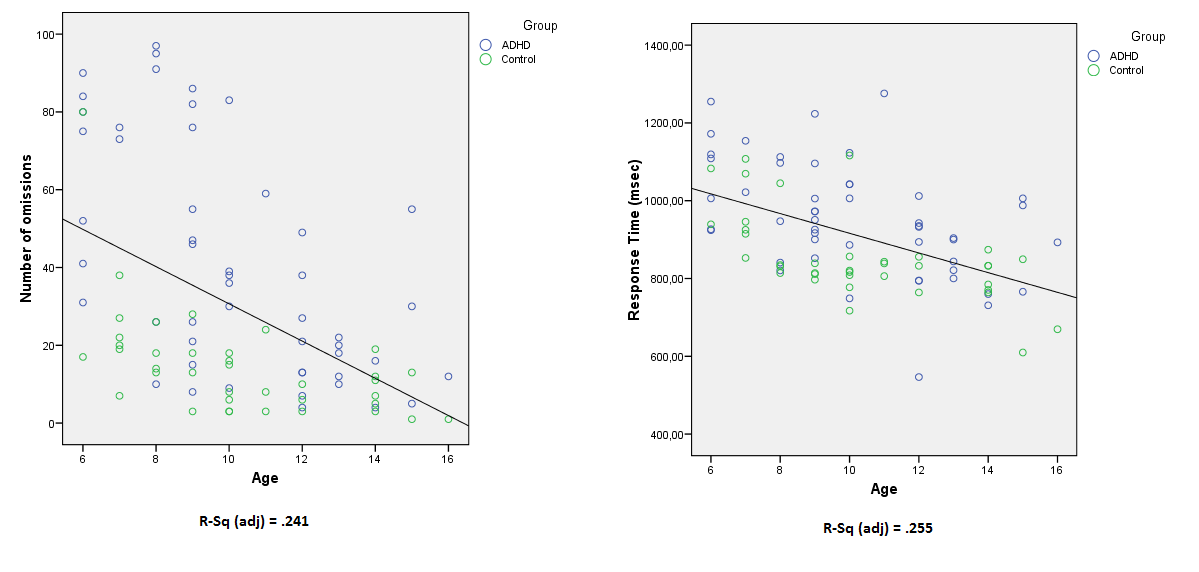

Supplement: S3 Fig — (TIF) [file pone.0201039.s003.tif]
